# Normative Computed Tomography Angiography Values of the Aortic Root, Aorta and Aortic arch in Children

**DOI:** 10.21203/rs.3.rs-4406785/v1

**Published:** 2024-05-17

**Authors:** Rakesh Donthula, Wen Li, Archita Duvvada, Dan Dyer, Santosh C. Uppu

**Affiliations:** the University of Texas McGovern Medical School at Houston; the University of Texas McGovern Medical School at Houston; The University of Texas at Austin; Rady Children’s Hospital; The University of Texas Health Science Center at Houston

**Keywords:** Computed Tomography Angiography, Z-scores, Normative values, Pediatrics, Aorta, Aortic root

## Abstract

**Purpose:**

Normative values for intracardiac and extracardiac vascular structures help in understanding normal growth and changes over time in children; this normative data are not currently available for ECG-gated Computed Tomography Angiography (CTA). We sought to establish ECG-gated CTA derived normative values for the aortic root, aorta and aortic arch in children.

**Methods and Results:**

Aortic root, ascending aorta, aortic arch, and descending aorta were measured in systole and diastole in 100 subjects who had ECG-gated CTA at our center between January 2015 through December 2020 and met our inclusion criteria. The allometric exponent (AE) for each parameter was derived, and the parameter/body surface area^AE^ (BSA^AE^) was established using the previously described methods. Using this data, normalized mean, cross-sectional area, and standard deviation were calculated. Z-score curves were plotted in relation to the BSA for all measurements.

**Conclusion:**

Our study reports systolic and diastolic ECG-gated CTA Z-scores along with normative curves in relation to BSA for the aortic root, aorta and aortic arch in children.

## Introduction

Overall survival and outcomes have improved in patients with congenital heart disease due to significant advances in the diagnosis, management, and treatment^1 2^. Cardiac imaging including echocardiography (echo), cardiac ECG-gated computed tomography angiography (CTA), and cardiac Magnetic resonance imaging (CMR) play a vital role in the diagnosis and follow-up of patients with congenital heart defects and patients with connective tissue disease (Marfan, Ehler-Danlos, Loeys-Dietz syndrome etc)^3 4 5 6 7^. Normative data for intracardiac and extracardiac vascular structures in the pediatric population are available for echo, CMR and non ECG-gated CTA^8 9 10 11^. As a result, imagers tend to interpret the CTA aortic measurements with known echo or CMR normative data, this is not ideal due to the inherent variation of the imaging modalities.

The utility of CTA is gaining acceptance in pediatric cardiology with improvements in scanning techniques, advances in radiation reduction, ease of access, and its ability to limit anesthesia exposure in young children due to fast scan times^6,12^. Cross-sectional imaging with CTA and CMR has added benefit in advanced planning for interventional procedures, creation of virtual or 3D models, overlaying with angiography to reduce radiation dose during an intervention, etc^13–16^. En face measurements using double oblique technique derived from cross-sectional imaging are made to report major and minor dimensions for a structure which negates the assumption that a vascular structure has a circular profile^17^.

Somatic growth should be considered for the pediatric population. For that reason, Z-scores are commonly used with standard deviations (SD) above and below a given measurement, but SDs are not constant across body sizes due to heteroscedasticity adding further complexity to the interpretation. Prior studies have attempted to report non-ECG gated CT diameters of aortic structures^18–20^. We have previously published normative ECG-gated CT Z-scores for the main and branch pulmonary arteries^21^. In this study, we sought to establish ECG-gated CTA-derived normative values and Z-scores for the aortic root and aorta in children.

## Methods

### Data Sources:

The study was approved by our institutional review board (The University of Texas Health Science Center at Houston and Memorial Hermann, (HSC-MS-20–1338, September 13^th,^ 2021) and waived the need for informed consent. We conducted a retrospective chart review including all consecutive patients less than 18 years of age who underwent ECG-gated- cardiac CTA between January 2015 and December 2020. We identified subjects using our imaging database and electronic medical records. Inpatient and outpatient records were analyzed to obtain baseline information.

### Population:

All children less than or equal to 18 years of age, who underwent ECG-gated cardiac CTA at our institution from January 2015 to December 2020 who met the inclusion criteria as listed in Table 1 were identified, those meeting the exclusion criteria were removed from the final analysis. The diagnosis and indication of the CTA for the included subjects are listed in Table 2. As cardiac CTA is not routinely performed in normal subjects, we broadly defined subjects without cardiac involvement as “normal”. Children with vascular rings (double aortic and right aortic arches) were also included in this study. For vascular ring subjects only the aortic root, ascending aorta and descending abdominal aortic measurements away from the vascular ring were recorded as normal, in this way we avoided the area of pathology. As such the number of subjects has been lower compared to an echo or CMR study with similar goals^22,23^.

### Patient selection, preparation, and contrast medium:

All patients were referred to our imaging center by a pediatric cardiologist for further evaluation. An initial screen was performed by an attending imaging cardiologist prior to cardiac CTA after reviewing echocardiographic information, patient medical records, and available clinical and diagnostic information. Those deemed candidates for CTA followed our institutional imaging protocol. Subjects younger than 6 weeks often underwent feed and swaddle techniques to minimize the need for sedation. Subjects between 6 weeks and 6 years were individualized based on the imaging goal, if anesthesia was needed, deep sedation without intubation was preferred for subjects with vascular rings. In subjects with a feed and swaddle approach image acquisition and timing were geared toward the clinical question. Some of these subjects had diagnostic studies but the image quality may not have been adequate for our study purpose as such they were excluded. Subjects less than 6 years requiring coronary artery evaluation were often anesthetized and intubated for better heart rate and respiratory control to avoid motion artifacts^24^. The location of the peripheral intravenous (PIV) access was determined by the imaging question. To evaluate for the aortic arch abnormalities, a foot PIV was preferred for contrast injection. Iso-osmolar, nonionic, and water-soluble agents (Iodixanol, Visipaque^™^ 320 mg iodine/ml) were used for all the examinations^25^. The total contrast dose was 1.5–2 ml/kg of body weight. The contrast medium was administered using a dual-head power injector at an injection rate of 0.8–1.2 ml/s for a 24G PIV. The injection rate is increased based on the size of the PIV up to 4–6.5 ml/s^12,26^. A bolus of isotonic saline solution was administered after contrast to reduce high-density contrast (streak) artifact. Automated bolus tracking technique was used with reference cardiovascular structure being monitored at near real-time until predetermined threshold opacification of 120–150 Hounsfield units was achieved at reference level. A scan delay of 4–5 seconds was often used for image acquisition^27^.

### Cardiac CTA technique:

During the study period, different generations of Siemens CT scanners were used to perform these studies. These include SOMATOM AS 128, SOMATOM definition edge, and SOMATOM Force Dual-source CT (Siemens Healthineers). Studies were performed according to our institutional protocol using a tube voltage of 70–100 kV and tube current auto modulation (CareDose4D, Siemens Healthineers), a slice thickness of 0.6 mm^9,26,28^. Retrospective electrocardiography (ECG)-gated acquisition was performed when ventricular volume/function and coronary artery evaluation were needed. The prospective ECG-triggered technique was used to delineate vascular anatomy^11^. Images were reconstructed with a slice thickness of 0.6 mm and an increment of 0.4–0.6 mm^29^.

Images acquired when the aortic valve was open were included under systole, usually between 30 and 40% RR interval. and the images acquired during 70–80% RR interval were included under diastole. Subjects who underwent retrospectively ECG-gated CTA had both systolic and diastolic images. Prospectively ECG-gated CTA images were acquired either in systole or diastole based on the subject’s heart rate as per the machine algorithm and imager preference to minimize motion artifact. All studies were performed with an imaging cardiologist present during the scan.

### Image Post-processing:

**Figure 1:**
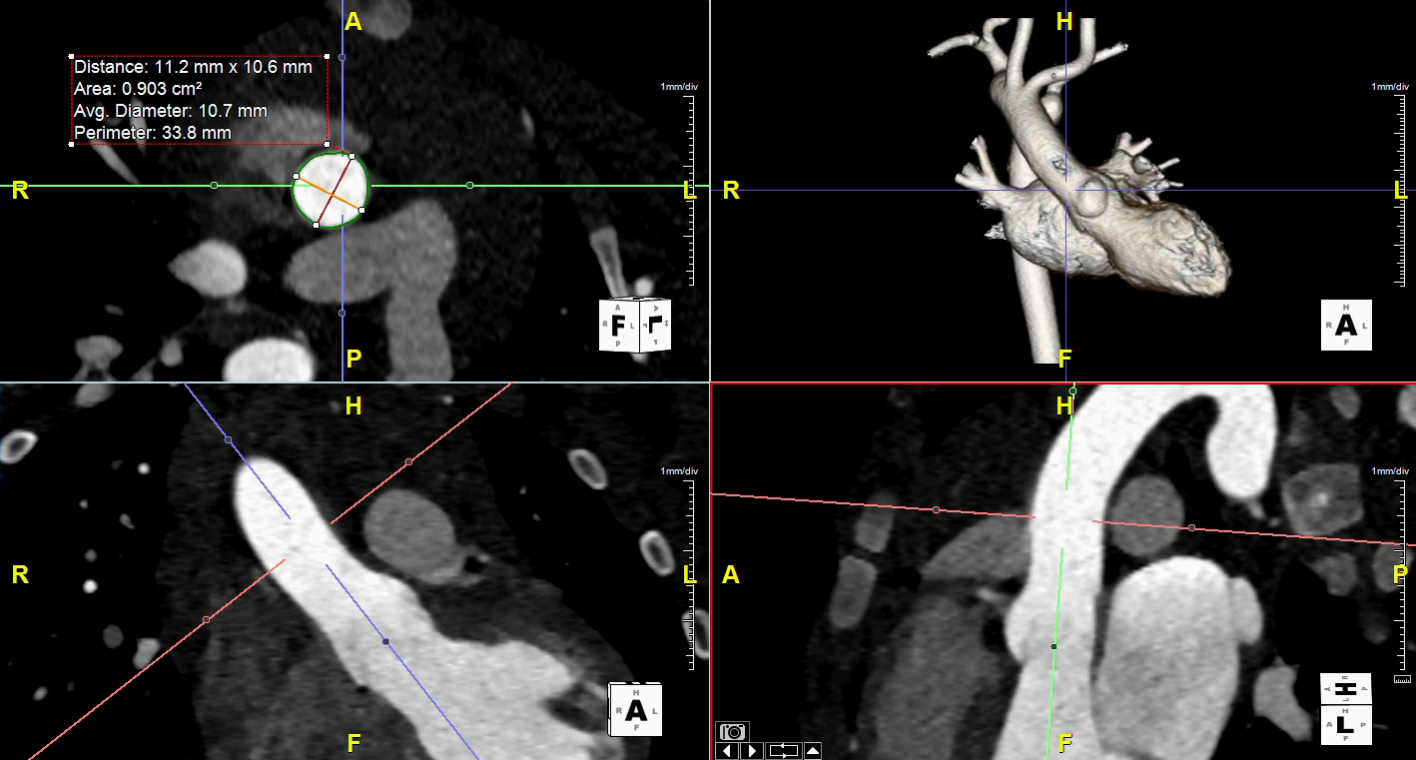
Example showing en face measurements of ascending aorta at the level of the pulmonary artery branching using the double oblique method.

**Figure 2:**
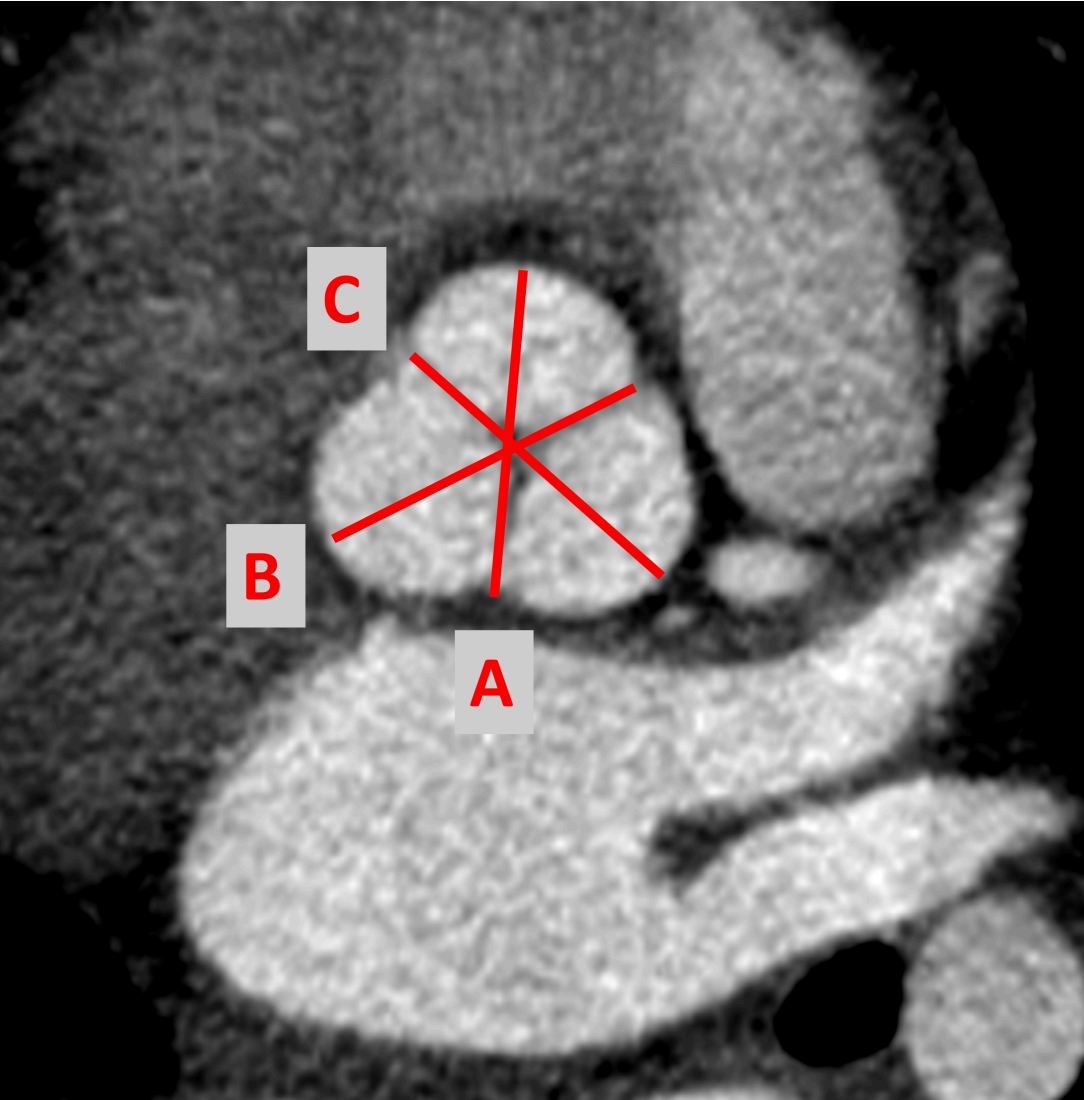
Example showing Aortic root measurements in diastole with the valve closed.

All measurements were performed on a cardiovascular imaging workstation (Circle CVI42; Circle Cardiovascular Imaging Inc. Alberta, Canada) after studies were anonymized. All images were visualized in axial, sagittal, and coronal planes with a window (600–900) and a level (250–350) settings. Double oblique planes using multiplanar reformatting were performed to measure aortic root, sinotubular junction (STJ), ascending aorta (AAO) at the level of the branch pulmonary artery, proximal (PTA) and distal transverse arch (DTA), aortic isthmus (IS), and descending aorta (DAO) at the level of the diaphragm^17,18,30^. As mentioned arch measurements for vascular ring subjects were not performed to avoid errors. Maximum, minimum, and mean diameters and cross-sectional area are measured for all the parameters except for the aortic root. (Fig. 1). For the aortic root, three measurements were made at mid- sinus level at their maximum dimensions. Cusps were named by the expected coronary artery origin, and commissures go along with them^31^. Aortic root measurements for this study are named as follows (Fig. 2).

- Right cusp to left/non-coronary commissure diameter
- Left cusp to non-coronary/right commissure diameter
- Non-coronary cusp to right/left commissure diameter.

The measurements were categorized as either systole or diastole as described above. Aortic measurements were performed at the following levels: ascending aorta was measured at the level of the right pulmonary artery/bifurcation of pulmonary arteries^18,20^, proximal transverse arch was measured immediately after the first branch of aorta, distal transverse arch was measured before the left subclavian artery, isthmus was measured after the last aortic branch. Descending Aorta was measured at the level of the diaphragm^18^.

### Statistical analysis:

Data was expressed as mean ± SD. The measure of the vessel was indexed to the body surface area (BSA) according to the Haycock formula^32^. The allometric exponent for each parameter was derived by applying the ordinary least squares method in which the natural logarithm of the parameter was regressed on the natural logarithm of BSA^33,34^. This method allows for a nonlinear relationship between the parameter and BSA. Specifically, the following steps were used.

1. The equation *Y = mX*^b^ was considered to decide the potentially nonlinear relationship between a parameter and BSA. *Y* denoted the parameter, *X* denoted BSA, and *b* was the AE to be estimated.
2. After taking the natural log, a linear regression formula was obtained: *ln(Y) = ln(m) + bln(X)*, where *ln(Y)* was the dependent variable and *ln(X)* was the independent variable.
3. The regression coefficient estimate $\hat{b}$ for *b* from the least squares method was the derived AE. We then indexed the parameter using BSA to the power of the derived AE. We did the following quality check to make sure that the allometric model was adequate and that there was no residual relationship between the indexed parameter and BSA. The indexed parameter was regressed against BSA. The regression line was plotted, and *R*^2^ was calculated. A flat line and a small *R*^2^ indicate no residual relationship. Pearson correlation coefficient and the corresponding P value were determined. A correlation close to zero and a P-value ≥ 0.05 also indicate no residual relationship.

We checked the normality of the indexed parameter using the Shapiro–Wilk test and reported μ ± σwhere μ and σ denoted the mean and SD of the indexed parameter. Last, we plotted non-indexed parameters against BSA with lines representing the mean, ± 1, ± 2, and ± 3 SDs of the mean based on the relationship that the non-indexed parameter followed a normal distribution with a mean of μBSA^AE^ and SD of BSA^AE^. Interobserver and intraobserver variability were assessed using the intraclass correlation coefficient in a subset of 50 subjects. All analyses were conducted using R version 4.0.5 (March 2021).

## Results

### Patient characteristics:

Out of 628 patients who underwent CTA at our institution during the study period, 100 children met the inclusion criteria and were analyzed. The mean age and BSA of the subjects were 5.3 ± 6.1 years (range 0–18 years) and 0.8 ± 0.68 m^2^ (range 0.16–2.8 m^2^) respectively. Among the subjects, 56% were male (Table 3). The majority (71%) of our subjects were young with a BSA < 1 m^2^. The most common finding on the CTA was structurally normal heart (n = 29), followed by right aortic arch with aberrant left subclavian artery (n = 25), and coronary anomaly (n = 21) as shown in Table 2.

Systolic measurements for aortic root, STJ, AAO and DAO were available in the majority of our subjects (n > 77). Systolic IS and DTA measurements were available in 50 subjects. Systolic PTA measurement was available in very few subjects (n = 35) due to common origin of the innominate and left carotid artery (n = 15). The Diastolic measurements were available in fewer subjects (root = 37, STJ = 41, AAO = 47, PTA = 25, DTA = 34, IS = 34 and DAO = 49 respectively) due to the younger age of our study population the CTA imaging protocols are geared towards acquiring the image during the least heart motion to reduce artifacts. As such systolic imaging was common in young children due to fast heart rates.

### Normative values:

The normalized mean diameters (mm), cross-sectional area (mm^2^), and standard deviation in systole and diastole are shown in Tables 4, 5. Based on the results in these tables, we can calculate the Z-score of a measurement for a given BSA using the reported AE, mean, and SD of that parameter:

$$Z-\text{score}=\frac{\text{indexedvalue}-\mu }{\sigma },$$

where

$$\text{indexedvalue}=\frac{\text{measuredvalue}}{BSA^{AE}},$$

μ denotes the mean of the indexed parameter, and *σ* denotes the SD of the indexed parameter as described in the statistical analysis section. For example, if a subject has an Ascending Aorta Maximum in systole measurement of 17.44 mm and a BSA of 0.6 m^2^, we can derive the Z-score by plugging in the numbers (AE = 0.49, mean = 18.236, SD = 2.079) to the above equations. That is, $\text{indexedvalue}=\frac{17.44}{0.6^{0.49}}=22.40$, and $Z-\text{score}=\frac{22.40-18.236}{2.079}=2.00$.

**Figure 3:**
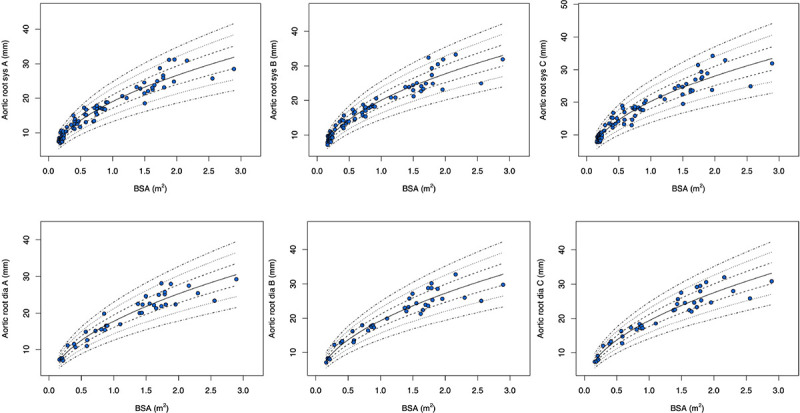
Regression lines for Z-score between − 3.0 and + 3.0 for nonindexed Aortic root diameters in systole and diastole. Sys- systole, Dia- diastole

**Figure 4:**
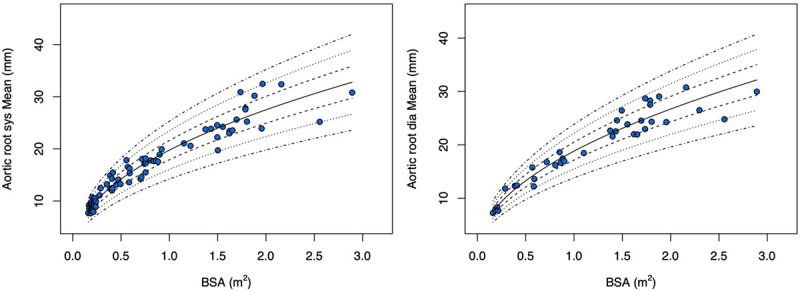
Regression lines for Z-score between − 3.0 and + 3.0 for nonindexed Aortic root mean diameters in systole and diastole. Sys- systole, Dia- diastole

**Figure 5:**
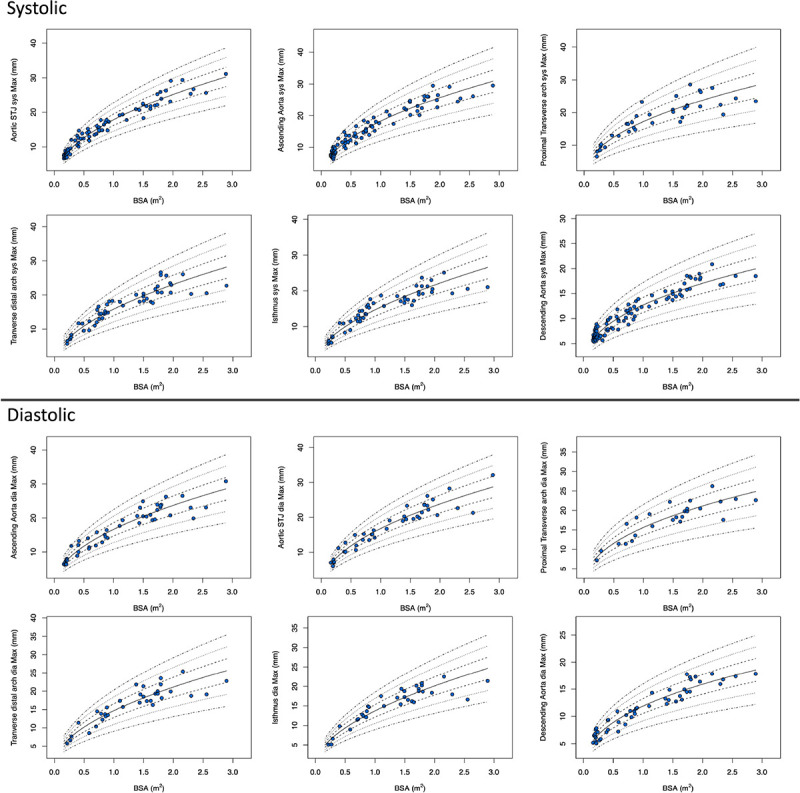
Regression lines for Z-score between − 3.0 and + 3.0 for nonindexed Aortic maximum diameters in systole and diastole. STJ - sinotubular junction, Sys- systole, Dia- diastole

**Figure 6:**
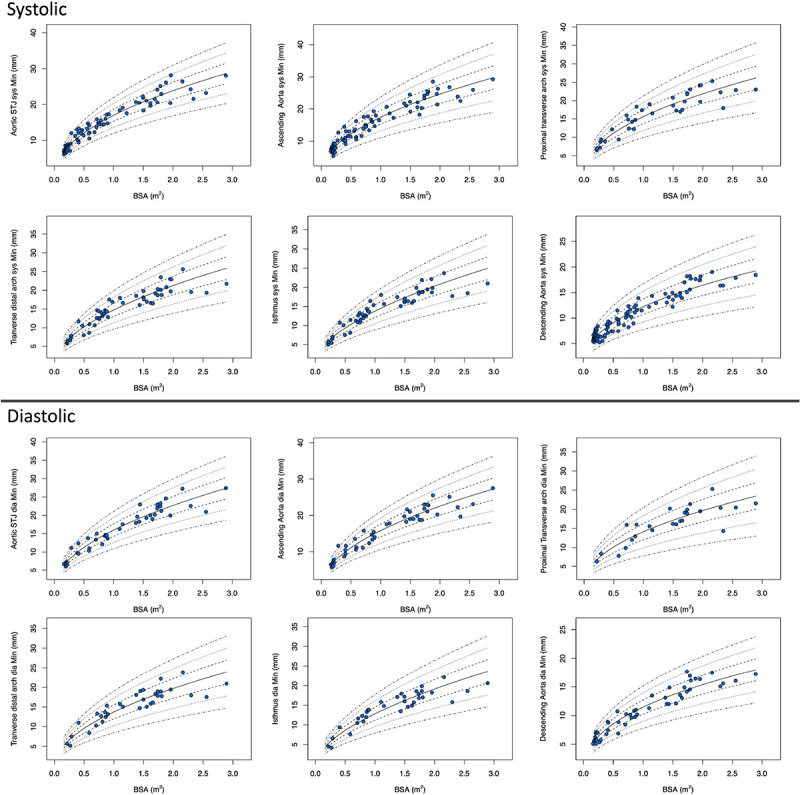
Regression lines for Z-score between − 3.0 and + 3.0 for nonindexed Aortic minimum diameters in systole and diastole. STJ - sinotubular junction, Sys- systole, Dia- diastole

**Figure 7:**
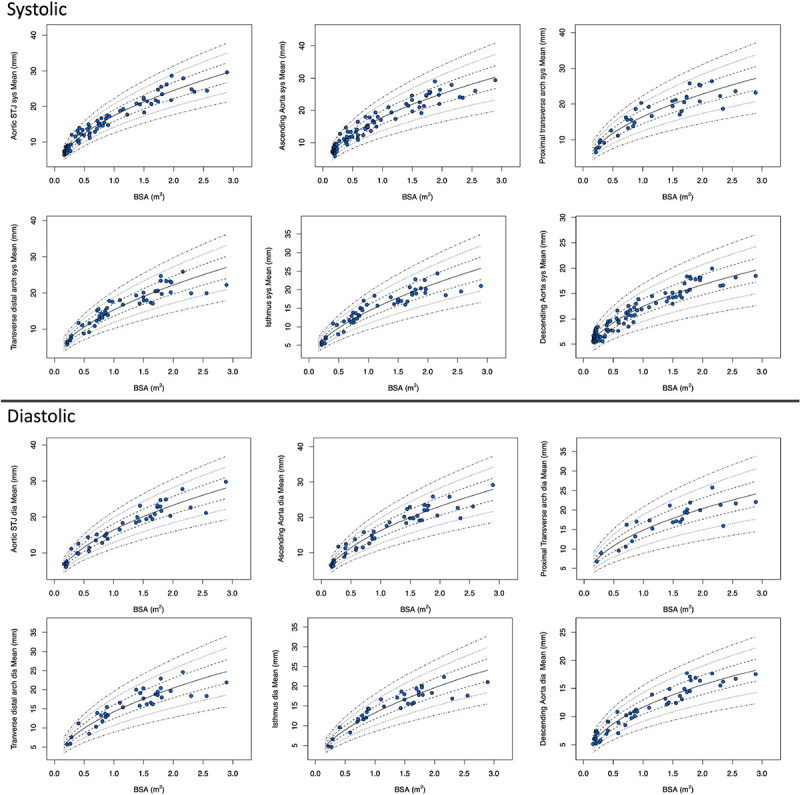
Regression lines for Z-score between − 3.0 and + 3.0 for nonindexed Aortic mean diameters in systole and diastole. STJ - sinotubular junction, Sys- systole, Dia- diastole

**Figure 8:**
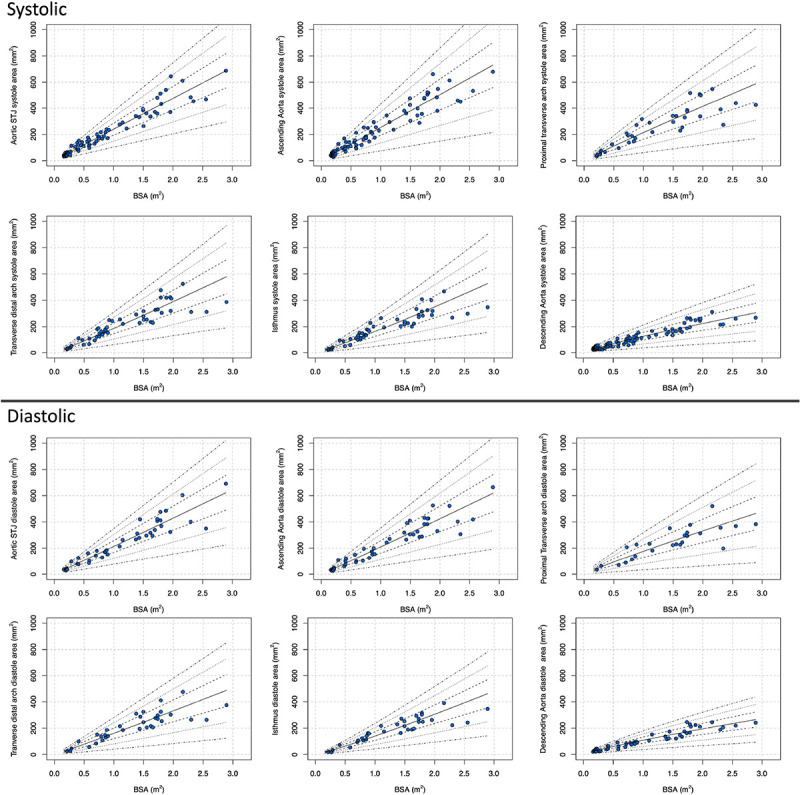
Regression lines for Z-score between − 3.0 and + 3.0 for nonindexed Aortic cross-sectional areas in systole and diastole. STJ - sinotubular junction, Sys- systole, Dia- diastole

The Z-score plots for systolic and diastolic aortic root diameters and their means are shown in Figs. 3, and 4. The Z-score plots for systolic and diastolic diameters, mean diameters and cross-sectional areas of the aortic structures are shown in Figs. 5, 6, 7 and 8 respectively. As expected systolic diameters were 6–11% larger and systolic cross-sectional areas were 13–20% larger compared to diastolic measures. Aortic root systolic measures were 5% larger than the diastolic measures.

### Reproducibility:

There was an excellent inter and intraobserver agreement (> 0.92) for both systolic and diastolic measurements (Tables 6, 7) indicating excellent reproducibility of our method.

## Discussion

We report normative systolic, diastolic diameters, cross sectional areas along with Z-scores and normative curves for the aorta, aortic root and aortic arch for ECG-gated cardiac CTA in children younger than 18 years. We report maximum, minimum, and mean diameters as well as the cross-sectional areas for these structures. Normative values in children exist for echo, CMR, and non ECG -gated CTA but are lacking for ECG-gated- CTA^35–37^. Few non-ECG-gated CTA studies reported normative measurements so far. Akay et al.^20^ used single axial measurement with respect to patient age, sex and T- vertebral body size. Bayindir et al.^19^ used single measurements from standard axial imaging planes as well. These measurements did not take the patient size into account; as such, the influence of growth and body size in a patient cannot be assessed. This methodology can also be challenging especially in patients with congenital heart defects, heterotaxy, etc. where a single-plane measurement may not be practical.

Hegde et al.^18^ reported aortic measurements using the double-oblique method using CTAs performed mostly in oncology, chest pain and trauma patients (42%, 16% and 10% respectively); their measurement technique was similar to our study. However, they have not reported cross sectional areas or used ECG-gated CTAs which the authors have recognized as a limitation. Their data was modeled using a natural log-transformed response variable.

En face measurements of the aorta provide an accurate assessment of the structure of interest^17^. Cardiac CTA is gaining acceptance in congenital cardiology to better evaluate vascular structures due to the improvements in scanning techniques, radiation reduction, ease of access, fast scan times, and reduced need for anesthesia in young children^6,28^.

Our study is also unique in reporting both systolic and diastolic normative aortic values; our study confirms larger systolic parameters compared with diastolic measures as was previously described by an angiographic study^38^.

The cross-sectional area along with the mean diameter may have real-world applicability as the structure of interest can be assessed in any non-standard plane. This is especially important in patients with congenital heart defects where a vascular structure may not be in a normal orientation.

Deriving normative values and Z-scores can be achieved by various methods and there is significant variation among Z-scores derived from each method^39–42^. We chose the previously described and validated method of using BSA as an expression of body size and linear regression for the relationship between body growth and cardiovascular dimensions^43,44 21,33,45–47^. Cardiovascular allometry is defined as the relative growth of cardiac structure in relation to somatic growth. Identifying a correct allometric relationship and model for a structure is important for its interpretation and clinical application^34,43^. Multiple authors have advocated the use of an allometric model to identify a true indexing method of a physiologically dependent variable and this method has been widely used for various echo Z-score calculators that are in current clinical use^33–35,43,44^.

Recruiting normal children for a CTA study is ethically not possible due to radiation exposure, need for sedation/anesthesia, and contrast use. It is not uncommon to identify normal subjects who undergo cross-sectional imaging for various reasons to be included in a study. Prior CMR and CTA studies to derive normative values for the aorta and pulmonary arteries have included children with a history of malignancy^22,23^. We have been diligent to include subjects with normal cardiac anatomy, as a result, this data should be close to what is expected for a normal population. Future studies are needed to validate our results with a larger sample. We plan to make our data publicly available.

### Limitations:

Our single-center, small-sample, retrospective study has inherent limitations due to its design. Generalized applicability across institutions may not be possible, but there is a lack of normative ECG-gated-CTA data available at this time. Our data will help kick-start the process of performing a similar study in a multicenter fashion which can help address issues related to a single-center design. Similar goals have been achieved by the Pediatric Heart Network echocardiogram database that published echocardiographic Z-scores, although normative echo Z-scores have been in existence for a few decades. The statistical methodology may have issues with the model of choice, there has been extensive literature on various Z-score models but we felt that our approach is ideal for this study based on extensive prior experience using allometric models in echocardiographic Z-score studies. Our study population was mostly young with 71% having a BSA of less than 1 m^2^, this resulted in less subjects available for the diastolic measurements as the CTA protocols were geared to acquire during systole. We have not reported aortic annulus, descending thoracic aortic measurements, we feel that the abdominal aortic measurements have more clinical significance. Our study has not explored the influence of sex, or race on the Z-scores due to the small sample size, a multicenter design is likely to address this issue. Our sample has predominantly young children, its applicability to older children and adolescents should be done with caution.

## Conclusions

This is the first pediatric study to report en face normative diameters and cross-sectional areas of the aortic root, ascending aorta and aortic arch in systole and diastole derived using ECG-gated cardiac CTA. This data has potential application for proper diagnosis, risk stratification, surgical planning, and planning catheter-related interventions for children with congenital heart defects and connective tissue disorders. The real-world applicability of this tool needs careful validation.

## Figures and Tables

**Table 1:** Inclusion and exclusion criteria

Inclusion Criteria	Exclusion Criteria
Age ≤ 18 years of age Structurally normal heart Vascular ring Anomalous aortic origin of the coronary artery Small patent ductus arteriosus in age < 1-month Small patent foramen ovale	Any CHD other than listed in the inclusion criteria. Aortic valve disease Diagnosed connective tissue disorder (Marfan, Ehler-Danlos, etc.) Rheumatic heart disease Depressed ventricular function by echocardiogram Dilated cardiac chambers by echocardiogram Motor vehicle accident with cardiac involvement Motion artifact on the CT scan History of cardiac surgery/intervention

CHD- Congenital Heart Defect

**Table 2:** Diagnosis of included subjects.

Diagnosis	Number (Total N = 100)
Structurally normal heart	29
Anomalous Aortic origin of coronary arteries	RCA (16), LCA (3)
Double aortic arch	13
Right aortic arch with aberrant left subclavian artery	25
Kawasaki disease follow-up with no coronary involvement, normal function	3
Others	9

* RCA- Anomalous aortic origin of the right coronary artery, LCA- Anomalous aortic origin of the left coronary artery.

**Table 3:** Demographics

Sex (%)	Male (56%)	
BSA < 1 m^2^	71%	
	Mean (SD)	Median (range)
**Age**	5.3 yrs (6.1)	2.3 yrs (0–18)
**Weight**	26.2 Kg (31.4)	12.75 Kg (2.04–156)
**Height**	98 cm (47.8)	88.5 cm (44–187)
**BSA**	0.8 m^2^ (0.68)	0.6 m^2^ (0.16–2.8)

BSA - body surface area, SD - standard deviation

**Table 4:** Maximum, minimum, mean, and area variables with AE, SD in systole.

Systolic parameter	n	AE	mean	Standard Deviation
Ascending Aorta Max	88	0.49	18.236	2.079
Ascending Aorta Min	88	0.51	17.398	2.118
Ascending Aorta Mean	88	0.5	17.817	2.069
Ascending Aorta Area	88	1	252.409	59.336
Aortic STJ Max	79	0.5	17.842	1.649
Aortic STJ Min	79	0.49	16.952	1.674
Aortic STJ Mean	79	0.5	17.398	1.625
Aortic STJ Area	79	0.99	239.495	45.584
Aortic root A	77	0.49	19.021	1.929
Aortic root B	77	0.47	20.029	1.85
Aortic root C	77	0.47	20.221	2.151
Aortic root Mean	77	0.48	19.757	1.847
Descending Aorta Max	94	0.42	12.704	1.501
Descending Aorta Min	94	0.43	12.234	1.497
Descending Aorta Mean	94	0.43	12.469	1.48
Descending Aorta Area	94	0.85	123.719	29.275
Isthmus Max	50	0.56	14.706	1.777
Isthmus Min	50	0.56	13.795	1.637
Isthmus Mean	50	0.56	14.251	1.688
Isthmus Area	50	1.11	161.471	38.26
Proximal Transverse arch Max	35	0.47	17.233	2.348
Proximal transverse arch Min	35	0.49	15.625	1.898
Proximal transverse arch Mean	35	0.48	16.43	1.977
Proximal transverse arch Area	35	0.95	213.975	50.953
Distal Transverse arch Max	50	0.54	15.891	1.874
Distal Transverse arch Min	50	0.53	14.777	1.716
Distal Transverse arch Mean	50	0.53	15.334	1.725
Distal Transverse arch Area	50	1.07	186.513	41.694

Max - maximum diameter, Min - minimum diameter, Mean - mean diameter, area - cross-sectional area, N - number of subjects, AE - allometric exponent, SD- standard deviation. Max, Min, and Mean diameters are reported in millimeters, cross-sectional area is reported in square millimeters. STJ - sinotubular junction. Aortic root: A- Right cusp to left/non-coronary commissure, B- Left cusp to non-coronary/right commissure, C- Non-coronary cusp to right/left commissure.

**Table 5:** Maximum, minimum, mean, area variables with AE, SD in diastole.

Diastolic parameter	n	AE	mean	Standard Deviation
Ascending Aorta Max	47	0.51	16.646	1.945
Ascending Aorta Min	47	0.51	15.82	1.747
Ascending Aorta Mean	47	0.51	16.233	1.816
Ascending Aorta Area	47	1.02	209.257	48.028
Aortic STJ Max	41	0.51	16.773	1.79
Aortic STJ Min	41	0.51	15.979	1.696
Aortic STJ Mean	41	0.51	16.376	1.715
Aortic STJ Area	41	1.01	212.678	45.333
Aortic root A	37	0.51	17.759	1.746
Aortic root B	37	0.5	19.323	1.857
Aortic root C	37	0.51	19.416	1.785
Aortic root Mean	37	0.5	18.833	1.673
Descending Aorta Max	49	0.42	11.902	1.358
Descending Aorta Min	49	0.42	11.463	1.229
Descending Aorta Mean	49	0.42	11.682	1.271
Descending Aorta Area	49	0.84	108.346	23.556
Isthmus Max	34	0.55	13.71	1.595
Isthmus Min	34	0.57	12.809	1.636
Isthmus Mean	34	0.56	13.26	1.576
Isthmus Area	34	1.13	139.727	32.264
Proximal Transverse arch Max	25	0.44	15.624	1.967
Proximal Transverse arch Min	25	0.48	14.14	2.105
Proximal Transverse arch Mean	25	0.45	14.885	1.982
Proximal Transverse arch Area	25	0.91	176.293	47.642
Distal Transverse arch Max	34	0.52	14.687	1.871
Distal Transverse arch Min	34	0.52	13.744	1.761
Distal Transverse arch Mean	34	0.52	14.216	1.775
Distal Transverse arch Area	34	1.04	160.828	40.149

Max- maximum diameter, Min- minimum diameter, Mean - mean diameter, area - cross-sectional area, N- number of subjects, AE - allometric exponent, SD- standard deviation. Max, Min, and Mean diameters are reported in millimeters, cross-sectional area is reported in square millimeters. STJ - sinotubular junction, STJ - sinotubular junction. Aortic root: A- Right cusp to left/non-coronary commissure, B- Left cusp to non-coronary/right commissure, C- Non-coronary cusp to right/left commissure.

**Table 6:** Inter and intraobserver variability for parameters in systole.

Systole variables	intra-rater	95% CI	inter-rater	95% CI
Ascending Aorta Max	0.978	(0.947, 0.991)	0.983	(0.95, 0.994)
Ascending Aorta Min	0.989	(0.973, 0.995)	0.985	(0.956, 0.995)
Ascending Aorta Mean	0.99	(0.975, 0.996)	0.987	(0.964, 0.996)
Aortic STJ Max	0.989	(0.973, 0.996)	0.975	(0.927, 0.992)
Aortic STJ Min	0.987	(0.967, 0.995)	0.991	(0.974, 0.997)
Aortic STJ Mean	0.983	(0.957, 0.993)	0.988	(0.963, 0.996)
Aortic root A	0.984	(0.958, 0.994)	0.978	(0.936, 0.993)
Aortic root B	0.982	(0.953, 0.994)	0.982	(0.946, 0.994)
Aortic root C	0.991	(0.977, 0.997)	0.99	(0.971, 0.997)
Aortic root Mean	0.994	(0.983, 0.998)	0.99	(0.969, 0.997)
Descending Aorta Max	0.968	(0.923, 0.987)	0.975	(0.93, 0.992)
Descending Aorta Min	0.925	(0.819, 0.97)	0.978	(0.938, 0.992)
Descending Aorta Mean	0.97	(0.925, 0.988)	0.981	(0.946, 0.994)
Isthmus Max	0.995	(0.981, 0.999)	0.991	(0.945, 0.999)
Isthmus Min	0.989	(0.956, 0.997)	0.983	(0.903, 0.998)
Isthmus Mean	0.996	(0.985, 0.999)	0.991	(0.948, 0.999)
Proximal Transverse arch Max	0.92	(0.599, 0.988)	0.895	(0.287, 0.993)
Proximal transverse arch Min	0.986	(0.92, 0.998)	0.983	(0.843, 0.999)
Proximal transverse arch Mean	0.967	(0.819, 0.995)	0.956	(0.634, 0.997)
Distal Transverse arch Max	0.967	(0.871, 0.992)	0.964	(0.803, 0.995)
Distal Transverse arch Min	0.984	(0.934, 0.996)	0.983	(0.904, 0.998)
Distal Transverse arch Mean	0.978	(0.913, 0.995)	0.977	(0.868, 0.997)

Max- maximum diameter, Min- minimum diameter, Mean - mean diameter, STJ - sinotubular junction, STJ - sinotubular junction. Aortic root: A- Right cusp to left/non-coronary commissure, B- Left cusp to non-coronary/right commissure, C- Non-coronary cusp to right/left commissure. CI - confidence interval.

**Table 7:** Inter and intraobserver variability for parameters in diastole.

Diastole variables	intra-rater	95% CI	inter-rater	95% CI
Ascending Aorta Max	0.995	(0.983, 0.999)	0.986	(0.952, 0.996)
Ascending Aorta Min	0.986	(0.955, 0.996)	0.987	(0.957, 0.997)
Ascending Aorta Mean	0.994	(0.979, 0.998)	0.993	(0.975, 0.998)
Aortic STJ Max	0.98	(0.925, 0.995)	0.969	(0.88, 0.993)
Aortic STJ Min	0.988	(0.956, 0.997)	0.979	(0.918, 0.995)
Aortic STJ Mean	0.988	(0.955, 0.997)	0.981	(0.923, 0.996)
Aortic root A	0.966	(0.838, 0.994)	0.94	(0.688, 0.991)
Aortic root B	0.968	(0.849, 0.994)	0.965	(0.808, 0.995)
Aortic root C	0.986	(0.93, 0.998)	0.976	(0.863, 0.996)
Aortic root Mean	0.98	(0.904, 0.997)	0.971	(0.836, 0.996)
Descending Aorta Max	0.91	(0.715, 0.975)	0.868	(0.579, 0.965)
Descending Aorta Min	0.959	(0.862, 0.989)	0.948	(0.817, 0.987)
Descending Aorta Mean	0.943	(0.813, 0.984)	0.922	(0.734, 0.98)
Isthmus Max	0.983	(0.903, 0.998)	0.981	(0.869, 0.998)
Isthmus Min	0.982	(0.899, 0.997)	0.979	(0.851, 0.998)
Isthmus Mean	0.983	(0.903, 0.998)	0.984	(0.889, 0.998)
Proximal Transverse arch Max	0.949	(0.677, 0.994)	0.961	(0.666, 0.997)
Proximal Transverse arch Min	0.982	(0.877, 0.998)	0.986	(0.87, 0.999)
Proximal Transverse arch Mean	0.969	(0.792, 0.997)	0.981	(0.825, 0.999)
Distal Transverse arch Max	0.985	(0.913, 0.998)	0.997	(0.975, 1)
Distal Transverse arch Min	0.994	(0.962, 0.999)	0.996	(0.969, 1)
Distal Transverse arch Mean	0.996	(0.975, 0.999)	0.997	(0.975, 1)

Max- maximum diameter, Min- minimum diameter, Mean - mean diameter, STJ - sinotubular junction, STJ - sinotubular junction. Aortic root: A- Right cusp to left/non-coronary commissure, B- Left cusp to non-coronary/right commissure, C- Non-coronary cusp to right/left commissure. CI - confidence interval.
